# Electromyographic Assessment of Masseter Muscle Activity: A Proposal for a 24 h Recording Device with Preliminary Data

**DOI:** 10.3390/jcm12010247

**Published:** 2022-12-29

**Authors:** Anna Colonna, Lorenzo Noveri, Marco Ferrari, Alessandro Bracci, Daniele Manfredini

**Affiliations:** 1 Department of Biomedical Technologies, School of Dentistry, University of Siena, 53100 Siena, Italy; 2 Department of Neurosciences, School of Dentistry, University of Padova, 35128 Padova, Italy

**Keywords:** awake bruxism, sleep bruxism, bruxism, masseter muscle activity, electromyographic assessment, diagnosis

## Abstract

Objective: The instrumental measurement of electromyographic (EMG) activity in the natural environment is the best strategy available to collect information on bruxism. The twofold aim of this study was to (1) introduce and discuss a novel EMG device for the assessment of awake (AB) and sleep bruxism (SB) in the home environment over 24 h and (2) present some preliminary data. Methods: Fifteen healthy volunteers (eight males and seven females; mean age: 48.2 ± 4.1 years) underwent 24 h EMG recording trials of their masseter muscle activity (MMA) with a miniaturized wireless device. This device allowed us to measure the durations of the different phases of the recordings (total duration, awake time, sleep time, and electrode dislodgement time) as well as the bruxism time index (BTI) and bruxism work index (BWI) for both the waking and sleeping hours. Results: For the healthy volunteers, on average, the bruxism work index (BWI) values were 0.4 ± 0.2 and 0.1 ± 0.1 for awake and sleep, respectively, while the mean bruxism time index (BTI) values were 0.9 ± 0.5 for awake and 0.3 ± 0.1 for sleep. Conclusions: This investigation describes the technical features of a novel EMG recording device that permits the evaluation of masseter muscle activity in the home environment over 24 h. For the first time, a dedicated elaboration of the EMG signal allowed an assessment of muscle work and not just a count of purported SB/EMG events. Clinical significance: Based on the multidisciplinary approach in the study of bruxism, such a methodology, thanks to its peculiar features, will allow researchers and clinicians to monitor the epidemiology of MMA and delve deeper into the awake and sleep bruxism correlates for tailored management in clinical settings.

## 1. Introduction

Bruxism is an oral condition that is gaining attention from clinicians and researchers in several medical fields, such as orofacial pain experts, dentists, neurologists, physicians, and psychologists. The constantly evolving knowledge and the different bruxism constructs adopted by the various specialties are reflected in the number of different definitions that have been provided over the past decades [1,2]. Currently, an expert consensus definition has finally been embraced, providing separate definitions for sleep bruxism (SB) and awake bruxism (AB).

Sleep bruxism is a masticatory muscle activity during sleep that is characterized as rhythmic (phasic) or nonrhythmic (tonic) and is not a movement disorder or a sleep disorder in otherwise healthy individuals.

Awake bruxism is a masticatory muscle activity during wakefulness that is characterized by repetitive or sustained tooth contact and/or bracing or thrusting of the mandible and is not a movement disorder in otherwise healthy individuals [3].

As underlined by a recent literature review, both definitions start with ‘masticatory muscle activity’ (MMA), a wording intended to emphasize that focus is put on motor phenomena independent from any definite neurological correlates [1]. In addition, based on an appraisal of the advantages and limitations of the available diagnostic approaches, the international expert consensus panel originally proposed a diagnostic grading system for the operationalization of bruxism diagnosis [1,3], which was then upgraded to the ongoing project of defining a standardized tool for the assessment of bruxism (STAB) [4]. The basic premise of the STAB is that as much data as possible should be gathered on bruxism status. As such, the instrumental measurement of electromyographic (EMG) activity in the natural environment is the best strategy available to collect information on bruxism as an MMA [5,6].

Several home EMG recording devices have been introduced over the past few years to detect SB episodes [7,8,9] as alternatives to the more technically demanding polysomnography (PSG). On the other hand, AB EMG recording has never been performed routinely due to the scarcity of dedicated devices on the market, which also proved to be difficult to use due to their underwhelming designs [10,11,12].

In addition, there is a need to define and standardize some technical and conceptual aspects to implement the spectrum of measurements with respect to the simple count of EMG peaks over a certain threshold (i.e., the current PSG/SB criteria) [13,14]. Evaluating the amount of muscle work is a promising strategy for a better understanding of the clinical correlates [15].

Within this framework, a novel miniaturized electromyographic (EMG) recording device has been conceptualized to assess masseter muscle activity in the home environment over 24 h. This manuscript aims to describe the technical features of the device as well as present some preliminary data and the potential clinical implications of its use.

## 2. Materials and Methods

This study was performed on a sample of otherwise healthy adults who were recruited among the dental patients at the University of Siena, Siena, Italy. The research protocol was approved by the Institutional Review Board of the Orofacial Pain Unit, University Siena, Siena, Italy. All individuals gave their informed consent, in accordance with the Declaration of Helsinki, and understood that they were free to withdraw from the study at any time.

**Figure 1:**
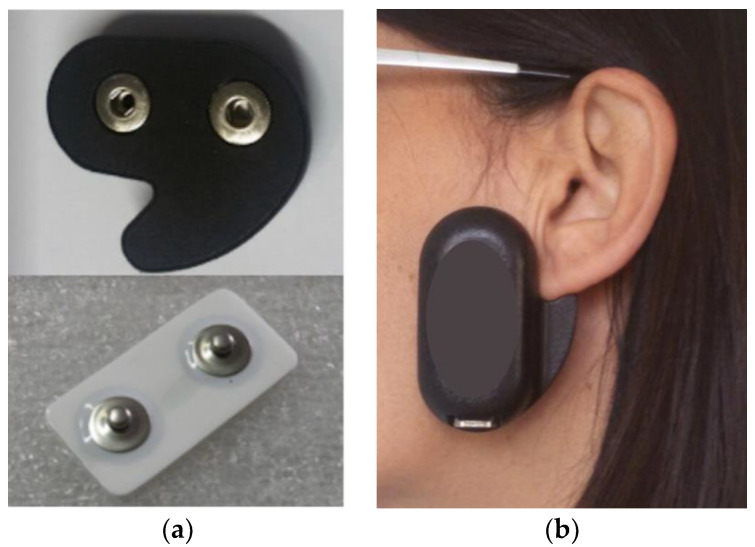
(**a**,**b**) Device used in this investigation.

Nineteen subjects underwent consecutive 24 h EMG recording trials of their masseter muscle activity with a miniaturized wireless device attached on the skin overlying the left masseter (dia-BRUXO, Biotech-Novations, Sanremo, Italy) (Figure 1a,b).

The device embeds an electronic circuit made of a single highly sensitive amplifier that is designed to measure the weakest electrical signals of EMG applications. Signals are detected by means of disposable bipolar electrodes with solid gel, AgAgCl sensors, and an interelectrode distance of 22 mm. The particular shape guides the correct positioning and the corresponding orientation of the electrodes in correspondence with the left masseter muscle, thus ensuring the repeatability of the position in the same patient in all subsequent recordings (Figure 1a,b).

The detected signal is then processed by a three-stage analog circuit: an amplification circuit, an active bandpass filter (between 110 Hz and 550 Hz), and an RMS (root-mean-square) integrator, which finally adapts it for acquisition within a high-performance processor. The analog information is digitalized in the processor by a 12-bit analog/digital converter (4096 discriminating levels), with an acquisition every 100 mS. The acquired signal can be treated in several ways as far as its interpretation, processing, and storage are concerned.

**Figure 2:**
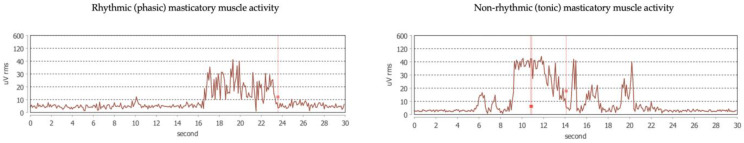
Example traces of different bruxism activities.

**Figure 3:**
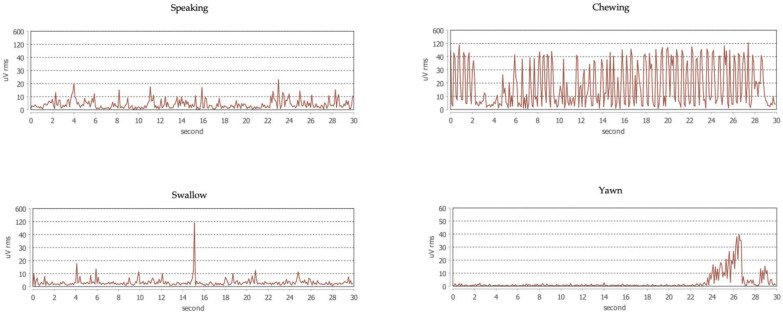
Example traces of physiological activities of the masseter muscle.

The EMG signal is interpreted by automatic scoring via a dedicated software based on an analysis of the continuous trace. The software is under testing to recognize and discriminate the different bruxism activities (Figure 2) as well as the physiological (i.e., functional) muscle activities, such as swallowing, chewing, speaking, and yawning (Figure 3). Before downloading the data, the patient is asked to set the time of falling asleep for software recognition.

In detail, each recorded physiological activity has been isolated and has been coded through algorithms useful for identifying it. The other activities have been classified as “Possible bruxisms”, and thanks to further algorithms the tonic and phasic activities have been isolated. The remaining recorded activities (not identifiable as physiological activities or bruxism) are indicated as “Other”.

In summary, the dedicated software excludes all the activities considered to be physiological or classified as “Other” from the calculations, taking only the tonic and phasic muscular activities into consideration.

This is a project under development, and for this reason the algorithms are in the process of obtaining a patent and cannot yet be disclosed.

The main parameters are the evaluation of the bruxism work index (BWI) and the bruxism time index (BTI). 

In detail, the bruxism work index (BWI) is defined as the percentage of muscle work during bruxism-related MMA compared to the potential work that could be exerted if the highest peak of power registered during the 24 hours had been kept unvaried during all the bruxism episodes. It is calculated with respect to its maximum recorded value, multiplied for the duration of all bruxism events:

$$\mathrm{BWI}=\frac{100*t\sum_{i=1}^{n}x_{i}k_{i}}{\sum_{i=1}^{n}x_{max}k_{i}}$$

*x_i_*: EMG sample value (in µV); *x_max_*: EMG maximum recorded value (in µV).

The bruxism time index (BTI) is defined as the percentage of time with bruxism-related MMA with respect to the total recording time.

## 3. Results

Of the 19 candidate volunteers, 4 were not eligible for the study because of a history of TMD pain (N = 2) or the presence of systemic rheumatic disease (N = 2). This led to a final sample of 15 participants (8 males and 7 females; mean age: 48.2 ± 4.1 years) taking part to the study.

Descriptive statistics for each variable, expressed as mean values, standard deviations, minimums, and maximums, are presented in Table 1 and Table 2.

For the fifteen healthy volunteers, the mean total recording time was 23.9 ± 0.5 h; in detail, the sleep duration was 8.4 ± 0.7 h during the recording night, and no considerable sleep interruptions that might have influenced the outcomes were reported by any subjects, while the awake recording duration was 15.5 ± 1.2 h. The electrode dislodgement time was, on average, 26.2 ± 58.5 min per patient, with a minimum of 0 min and a maximum of 200 over the 24 h EMG recording (Table 1).

On average, the awake BTI and BWI were 0.9 ± 0.5 and 0.4 ± 0.2, respectively, while the mean BTI and BWI for sleep were 0.3 ± 0.1 and 0.1 ± 0.1 (Table 2).

## 4. Discussion

Bruxism has a non-negligible prevalence (rates among adults range from 22% to 30% for AB and from 8% to 15% for SB) [16], creating new chairside concerns for dentists. In fact, bruxism represents a possible risk factor for clinical consequences such as pain in the jaw muscles or the temporomandibular joints (TMJ), severe tooth wear, repeated fractures, failure of dental restoration, and prosthodontic complications [1,2,3]. In addition, from an etiological viewpoint, bruxism may also be the mirror of underlying conditions, thus requiring the dentist to act as a sentinel for general health. For instance, gastroesophageal reflux may occur in SB patients, who may engage in jaw muscle activity to reduce the risk of detrimental chemical tooth wear by increasing salivation [1,2,17]. Moreover, a certain number of bruxism episodes can occur in correspondence with the end of respiratory arousals, possibly being instrumental to restore the patency of the upper airway while asleep [18]. However, the existence of an association between SB and sleep disorders is controversial, and the mechanism underlying this association is still not entirely clear [18,19,20]. Moreover, some kinds of bruxism activities may resemble a need to discharge emotional tension [21]. Thus, the exact clinical relevance of bruxism has yet to be determined, and epidemiological studies to investigate the biological variability in otherwise healthy individuals are needed as a starting point for future comparisons.

Recently, given the complexity of this phenomenon, the construct of bruxism has been extended to include a wider spectrum of jaw-muscle activities than provided in the past. This means that PSG/SB criteria, which are based on the count of masseter EMG events associated with sleep arousals, may provide only a partial picture of the complex range of jaw-muscle activities, especially considering that long-lasting, prolonged, low-activity contractions are now incorporated within the bruxism definition. In addition to that, definitive criteria have never been established for awake bruxism measurement. In view of this, the expert consensus panel pointed out the need to evaluate this phenomenon in its continuum [1,2,4,22]. The current knowledge is mostly related to SB, while there is a paucity of data on AB’s prevalence and natural course. This assumes importance in light of the fact that most of the data are drawn from retrospective self-reports at single observation points, and ecological assessment strategies were only recently introduced in the bruxism field [23,24].

Within these premises, this manuscript has introduced a novel miniaturized electromyographic (EMG) recording device that can be used to safely monitor masseter muscle activity over the full 24 h span and present some preliminary data. This type of device (i.e., home EMG recording) has some advantages with respect to the full audio–video PSG and EMG recordings that have been proposed for the AB monitoring regarding costs, design (e.g., footprint), and simplicity [10,11,12], and it is therefore a viable option for the study of sleep and awake bruxism on a larger scale [25]. Moreover, to our knowledge, this is the first device of its kind, allowing an evaluation of MMA during both sleep and wakefulness for the first time in its continuum thanks to a dedicated elaboration of the EMG signal that is not just based on the count of purported SB/EMG events. In fact, as suggested by recent articles, the theoretical concept of a cut-point for bruxism as a risk factor for any precise consequence is not biologically supportable. The effects depend on the type and amount of muscle work and the host response in both SB and AB. For this reason, studies should preferably be based on the measurement of the amount of bruxism behavior that increases or reduces the probability of any health outcomes [4,5].

The data gathered from the fifteen healthy adult volunteers who took part to this pilot investigation showed that, on average, the awake bruxism time index and bruxism work index were 0.9 ± 0.5 and 0.4 ± 0.2, respectively, while the mean BTI and BWI during sleep were 0.3 ± 0.1 and 0.1 ± 0.1. These data are not comparable with any other investigations since there is a paucity of works trying to measure bruxism in its continuum [15,26,27].

Although no evaluation of bruxism has been made in its continuum, only with regard to masseter activity peaks, it is interesting to note that Miyamoto et al. evaluated the biting function during the entire day in young adults by measuring the masseter muscle activity and found that the means of the number and the total duration of bursts over level 1 during the entire day were 7081 and 1668 s for the male subjects and 8922 and 2268 s for the female subjects, while the number and the total duration of high-amplitude bursts over levels 2, 3, and 4 during the entire day were substantially less than those of such low-amplitude bursts as level 1 [10]. 

The potential advantages of using this strategy in both the clinical and research settings are quite intuitive and may help overcome current difficulties in studying such phenomena. For researchers, this approach can be used to increase the knowledge on several aspects of sleep and awake bruxism, including the natural course and fluctuations in signs and/or symptoms and the exposure to etiological factors; on the other hand, a remarkable amount of data is going to be available to study the epidemiology of the various items at the individual (e.g., case series) and population levels (e.g., cross-sectional and longitudinal large-sample studies). Moreover, these data might be used to standardize future reports for comparison purposes. As an important clinical implication, the data could be useful to monitor sleep and awake bruxism evolution over time and help clinicians prevent and manage the possible consequences at the individual level.

The pathway toward routine use provides the need to validate any discrimination strategy between functional and nonfunctional activities as well as to assess the accuracy of the device with respect to the available standard of reference [28,29,30]. Nonetheless, the advantage of elaborating the signal as the expression of the potential amount of muscle work exerted during 24 h deserves further attention as the best strategy available to delve deeper into the study of MMA correlates to understand its relationship with specific clinical outcomes [3,5,31,32].

## 5. Conclusions

This investigation presented pilot data on a new device that, along with the identification of the EMG masseter activity peaks that are commonly used to define SB episodes, allows an evaluation of the full spectrum of muscle activities (i.e., MMA) that are currently included under the bruxism definition umbrella. This strategy opens the door to a new era for the evaluation of bruxism correlates, which was hampered until now by the difficulties in including an evaluation of the muscle work in the EMG signal processing. This methodology will allow researchers and clinicians to evaluate awake and sleep bruxism with a wide-ranging look at the clinical impacts of the different bruxism activities.

## Figures and Tables

**Table 1:** Descriptive statistics of outcome variables over the 24 h EMG recording (mean values; standard deviations; min; and max).

Outcome Variable	Average Values	SD	Min	Max
Total recording time (h)	23.9	0.5	22	24
Sleep time (h)	8.4	0.7	7	9
Awake time (h)	15.5	1.2	13	17
Electrode dislodgment time (min)	26.2	58.5	0	200

**Table 2:** Descriptive statistics of outcome variables over the 24 h EMG recording (mean values; standard deviations; min; and max).

Outcome Variable	Average Values	SD	Min	Max
A-BWI	0.4	0.2	0.2	0.9
S-BWI	0.1	0.1	0.0	0.3
A-BTI	0.9	0.5	0.3	2.1
S-BTI	0.3	0.1	0.1	0.5
